# Supplementary material for: Chemosensory Characteristics of Brandies from Chinese Core Production Area and First Insights into Their Differences from Cognac
Source: Foods. 2023 Dec 20;13(1):27. doi: 10.3390/foods13010027 (PMC10777998; doi:10.3390/foods13010027)
Supplement: Supplementary file 1 [file foods-13-00027-s001.zip › foods-2728616-supplementary.pdf]

Supplementary Table 1 Information for the Calibration Curve of Each Compound

No.	Compounds	CAS.	Quantitative ion（m/z）	calibration curve	R^2	Concentration range (μg/L)
1	ethyl acetate	141-78-6	-	y = 2.637x - 0.1479	0.9949	101750~13024000
2	2,3-butanedione	431-03-8	279	y=11.822x+0.0335	0.9971	7.8~16000
3	2-methyl-1-propanol	78-83-1	-	y = 0.6673x - 0.0797	0.9941	30454~7796160
4	2-methyl-1-butanol	137-32-6	57	y = 10.774x + 2.3865	0.9995	500-64000
5	3-methyl-1-butanol	123-51-3	-	y = 0.8299x - 0.1482	0.9975	13446~27536640
6	1-pentanol	71-41-0	42	y = 7.9791x + 1.638	0.9981	125~32000
7	3-methyl-3-buten-1-ol	763-32-6	41	y = 2.4801x + 2.2077	0.9937	125~32000
8	3-methyl-1-pentanol	589-35-5	56	y = 2.4374x - 0.9088	0.9936	125~32000
9	1-hexanol	111-27-3	56	y = 1.6767x + 0.2451	0.9994	1250~160000
10	ethyl lactate	97-64-3	-	y = 0.3058x + 0.066	0.9911	3000~1690240
11	(*Z*)-3-hexen-1-ol	928-96-1	41	y = 1.9401x + 0.6607	0.9946	750~96000
12	ethyl octanoate	106-32-1	88	y = 0.0971x - 0.0283	0.9964	9.4~9600
13	acetic acid	64-19-7	43	y = 22.497x - 0.7797	0.9946	10000~160000
14	furfural	98-01-1	96	y = 1.4725x + 0.153	0.9933	62.5~160000
15	2-acetylfuran	1192-62-7	95	y = 1.0209x - 0.1065	0.9988	125~32000
16	benzaldehyde	100-52-7	106	y = 1.0562x + 0.2654	0.9960	250~32000
17	ethyl 2-hydroxy-4-methylpentanoate	10348-47-7	69	y = 1.2114x + 0.6428	0.9962	125~32000
18	propanoic acid	79-09-4	74	y = 24.033x - 0.5419	0.9956	1000~64000
19	2-methyl-propanoic acid	79-31-2	43	y = 2.1217x + 0.1182	0.9998	1000~64000
20	5-methylfurfural	620-02-0	110	y = 4.377x - 3.3585	0.9954	250~32000
21	ethyl levulate	539-88-8	43	y = 0.4653x - 0.0524	0.9998	100~12800
22	ethyl decanoate	110-38-3	88	y = 0.576x + 0.091	0.9992	125~64000
23	ethyl 2-furoate	614-99-3	95	y = 0.2813x + 0.4111	0.9938	15.6~32000
24	3-methylbutanoic acid	503-74-2	60	y = 5.331x - 0.6668	0.9977	500~16000
25	diethyl succinate	123-25-1	101	y = 0.8181x - 0.0072	0.9929	250~32000
26	*β*-damascenone	23726-93-4	69	y = 13.032x - 7.2015	0.9953	125~32000
27	ethyl dodecanoate	106-33-2	88	y = 0.4453x + 0.1651	0.9917	125~32000

No.	Compounds	CAS.	Quantitative ion（m/z）	calibration curve	R^2	Concentration range (μg/L)
28	hexanoic acid	142-62-1	60	y = 14.33x - 25.213	0.9915	75~9600
29	guaiacol	90-05-1	109	y = 1.807x + 0.3688	0.9990	62.5~32000
30	(*E*)-whiskeylactone	39638-67-0	99	y = 0.0045x - 0.0097	0.9969	2.79~1427.5
31	2-phenylethanol	60-12-8	91	y = 0.3866x + 0.0941	0.9982	750~96000
32	(*Z*)-whiskeylactone	55013-32-6	99	y = 0.0048x - 0.0099	0.9955	2.09~1072.5
33	phenol	108-95-2	94	y = 0.7211x - 0.0792	0.9992	15.6~32000
34	ethyl tetradecanoate	124-06-1	88	y = 0.4328x + 0.2815	0.9967	62.5~32000
35	octanoic acid	124-07-2	60	y = 4.159x - 0.049	0.9942	750~96000
36	γ-nonalactone	104-61-0	85	y = 0.0092x - 0.0333	0.9941	2.2~1107.6
37	2-methoxy-4-vinylphenol	7786-61-0	135	y = 1.305x - 1.1454	0.9943	37.5~9600
38	ethyl hexadecanoate	628-97-7	88	y = 0.3972x + 0.3586	0.9935	31.3~32000
39	syringol	91-10-1	154	y = 0.6866x + 0.1593	0.9963	15.6~32000
40	γ-dodecalactone	2305-05-7	85	y = 0.0025x + 0.0001	0.9960	0.86~442.5
41	5-hydroxymethylfurfural	67-47-0	97	y = 4.0789x + 0.1024	0.9943	20000~640000
42	vanillin	121-33-5	151	y = 1.2165x + 0.0535	0.9915	250~32000
43	ethyl vanillate	617-05-0	151	y = 0.3598x + 0.2478	0.9971	62.5~32000

Supplementary Table 2 Reference for Aroma Descriptors in Sensory Evaluation

No.	Aroma descriptor	Reference
1	alcohol	EtOH/H_2O (40:60; v/v) solution
2	dried fruit	Le Nez du Vin-almond
3	mushroom	Le Nez du Vin-mushroom
4	floral	Le Nez du Vin-rose
5	fruity	Le Nez du Vin-blackcurrant
6	caramel	Le Nez du Vin-caramel
7	spicy	Le Nez du Vin-cinnamon、clove
8	toasted	Le Nez du Vin-toast、smoky

Supplementary Table 3 Identification of Odor-active Compounds Detected by GC-O and GC–MS in Brandies

No.	Compounds	RI	RIL	CAS.	Aroma descriptor	Odor intensity								
						CVS	CX10	CX15	HVS	HXO	MVS	MXO	RVS	RXO
1	ethyl acetate	863	887	141-78-6	sour fruit	2.0	2.1	2.1	1.1	1.5	1.7	1.6	1.6	1.6
2	2,3-butanedione	956	977	431-03-8	milky	1.5	2.8	3.0	1.5	2.5	1.5	2.1	3.0	3.3
3	2-methyl-1-propanol	1109	1098	78-83-1	green	1.7	1.4	1.8	1.8	1.8	1.9	1.9	2.0	2.1
4	2-methyl-1-butanol	1200	1207	137-32-6	solvent	3.2	2.8	3.1	2.8	2.6	2.4	2.2	2.0	2.1
5	3-methyl-1-butanol	1222	1211	123-51-3	bitter, roasted hazelnut	4.0	3.9	4.2	4.2	3.9	3.7	3.7	3.9	3.7
6	1-pentanol	1263	1255	71-41-0	sweet fruit	1.0	1.6	-	-	-	1.9	2.0	2.1	-
7	3-methyl-3-buten-1-ol	1270	1247	763-32-6	sweet, caramel	1.9	2.1	-	2.0	1.9	2.0	2.1	-	-
8	3-methyl-1-pentanol	1324	1331	589-35-5	creamy, fruity	2.1	2.2	-	2.4	2.7	2.6	2.7	2.7	2.6
9	1-hexanol	1347	1357	111-27-3	gasoline, gas	2.9	2.9	3.3	3.4	3.2	3.0	3.3	3.2	3.2
10	ethyl lactate	1361	1345	97-64-3	sweet fruit	1.9	2.1	2.1	2.2	2.0	1.9	2.3	3.4	2.2
11	(Z)-3-hexen-1-ol	1381	1389	928-96-1	green apple	3.0	3.1	3.3	3.5	3.3	3.3	3.2	3.3	3.3
12	ethyl octanoate	1383	1440	106-32-1	sweet fruit	1.9	2.2	2.3	2.4	2.4	2.2	2.1	2.0	2.0
13	acetic acid	1455	1461	64-19-7	acetic acid	4.4	4.4	4.4	4.3	4.3	4.4	4.3	4.4	4.4
14	furfural	1461	1457	98-01-1	toasted	2.4	2.5	2.7	2.6	2.5	-	2.9	-	-
15	2-acetylfuran	1505	1498	1192-62-7	salty	1.1	1.5	1.5	1.4	1.7	1.5	1.5	1.7	1.6
16	benzaldehyde	1519	1528	100-52-7	fresh green	-	-	-	-	-	2.2	2.2	-	-
17	ethyl 2-hydroxy-4-methylpentanoate	1526	1545	10348-47-7	lilac buds	2.2	2.4	2.8	2.6	2.9	2.7	2.7	2.7	2.8
18	propanoic acid	1538	1535	79-09-4	sour, cheese	-	-	-	2.4	-	2.2	2.3	2.1	2.0

No.	Compounds	RI	RIL	CAS.	Aroma descriptor	Odor intensity								
						CVS	CX10	CX15	HVS	HXO	MVS	MXO	RVS	RXO
19	2-methyl-propanoic acid	1554	1564	79-31-2	sour	2.4	2.5	2.7	2.9	2.7	2.9	2.9	2.6	2.8
20	5-methylfurfural	1573	1573	620-02-0	milk, sweet	2.3	2.3	2.2	2.1	2.2	2.2	2.1	1.9	1.9
21	ethyl levulate	1599	1614	539-88-8	sweet fruit	-	-	0.5	1.5	1.7	1.7	1.7	1.7	1.5
22	ethyl decanoate	1602	1633	110-38-3	apple	1.8	2.0	1.6	1.6	1.7	1.7	1.7	1.9	1.9
23	ethyl 2-furoate	1608	1611	614-99-3	toasted	2.5	1.9	1.8	2.0	1.9	1.7	1.8	2.1	2.2
24	3-methylbutanoic acid	1655	1664	503-74-2	sour	4.4	4.4	4.1	4.3	3.8	3.4	3.9	3.7	3.8
25	diethyl succinate	1655	1687	123-25-1	sweet fruit	2.2	2.1	2.4	2.7	2.7	2.7	2.7	2.7	2.8
26	*β*-damascenone	1821	1821	23726-93-4	sweet fruit	-	-	-	-	3.1	3.2	3.1	3.1	-
27	ethyl dodecanoate	1834	1843	106-33-2	black currant	3.7	3.7	-	3.7	3.9	3.9	3.7	3.8	3.7
28	hexanoic acid	1841	1850	142-62-1	bitter, acid	3.0	3.1	3.0	3.2	3.1	3.1	3.0	3.1	3.0
29	guaiacol	1861	1871	90-05-1	smoky	3.8	3.6	3.5	3.7	3.4	3.5	3.4	3.5	3.7
30	(*E*)-whiskeylactone	1900	1899	39638-67-0	caramel	3.0	3.4	4.0	3.3	3.9	3.5	3.5	3.4	3.8
31	2-phenylethanol	1910	1901	60-12-8	rose	4.4	4.2	4.3	4.3	4.2	4.4	4.2	4.4	4.5
32	(*Z*)-whiskeylactone	1968	1976	55013-32-6	toasted, caramel	3.2	3.2	4.2	3.6	4.0	3.1	3.8	2.9	4.1
33	phenol	1998	2004	108-95-2	sour	2.4	2.3	2.5	2.3	2.6	2.7	2.5	2.5	2.6
34	ethyl tetradecanoate	2037	2063	124-06-1	sweet fruit	-	-	-	-	-	-	-	2.9	2.7
35	octanoic acid	2044	2044	124-07-2	sour	2.9	2.8	-	2.9	2.6	2.6	2.7	2.7	2.7
36	*γ*-nonalactone	2046	2012	104-61-0	apricot, peach	1.8	2.0	2.0	3.0	3.5	3.8	3.0	2.6	4.0
37	2-methoxy-4-vinylphenol	2207	2194	7786-61-0	bitter, smoky	-	-	-	-	2.6	-	2.6	2.6	2.8
38	ethyl hexadecanoate	2229	2255	628-97-7	toasted, creamy	2.4	2.4	2.5	2.2	2.4	2.4	2.4	2.5	2.5

No.	Compounds	RI	RIL	CAS.	Aroma descriptor	Odor intensity								
						CVS	CX10	CX15	HVS	HXO	MVS	MXO	RVS	RXO
39	syringol	2278	2303	91-10-1	spicy, salty	3.6	3.6	3.5	3.9	3.8	3.9	3.8	3.6	3.6
40	γ-dodecalactone	2359	2376	2305-05-7	creamy	3.5	1.8	2.1	2.7	2.3	2.5	2.9	2.3	2.9
41	5-hydroxymethylfurfural	2519	2528	67-47-0	milky, sweet	2.0	1.9	1.9	2.0	2.1	2.2	2.1	2.0	1.9
42	vanillin	2595	2589	121-33-5	bitter, toasted	2.0	1.9	1.9	1.9	1.9	2.0	2.0	2.1	2.4
43	ethyl vanillate	2650	2668	617-05-0	smoky, bitter	1.9	2.2	1.7	1.7	2.1	2.2	2.2	2.2	2.1
44	methyl salicylate	1787	1778	119-36-8	fried peanuts	2.1	1.9	1.7	2.7	1.7	2.0			
45	creosol	1948	1959	93-51-6	bitter, medicinal	3.1	3.6	3.2	2.9	2.4	-	2.9	2.6	3.3
46	ethyl cinnamate	2092	2104	103-36-6	sweet				2.5	2.9	2.1	2.2	2.3	3.5
47	unknown	1099			toasted		2.2	1.7	2.1	1.9	1.8	1.8	2.2	2.9
48	unknown	1103			smoky					-	-	2.2	2.3	2.6
49	unknown	1186			caramel	1.9	2.9	2.7	3.2	3.1	2.5	2.7	2.4	3.3
50	unknown	2154			toasted	2.9	1.9	2.4	2.1	1.9	2.0	1.9	2.1	2.1
51	unknown	2319			spicy	3.5	3.4	3.4	4.0	3.1	3.9	3.8	3.6	3.8

Supplementary Table 4 **Variable Importance in the Projection (VIP) of the First Two Explanatory Variable of PLS-R Model**

Variable	VIP	Standard deviation	Lower bound(95%)	Upper bound(95%)	VIP	Standard deviation	Lower bound(95%)	Upper bound(95%)
5-methylfurfural	1.611	0.207	1.133	2.089	1.376	0.208	0.897	1.855
γ-nonalactone	1.593	0.239	1.043	2.144	1.378	0.193	0.932	1.825
ethyl lactate	1.526	0.438	0.516	2.535	1.327	0.374	0.463	2.190
γ-dodecalactone	1.503	0.618	0.077	2.929	1.348	0.512	0.167	2.529
ethyl 2-hydroxy-4-methylpentanoate	1.494	0.434	0.494	2.494	1.275	0.388	0.381	2.169
furfural	1.478	0.263	0.871	2.086	1.263	0.235	0.721	1.804
diethyl succinate	1.476	0.521	0.275	2.676	1.262	0.426	0.281	2.243
1-hexanol	1.349	0.524	0.140	2.558	1.152	0.469	0.070	2.234
octanoic acid	1.330	0.346	0.531	2.129	1.139	0.242	0.580	1.698
(Z)-whiskeylactone	1.312	0.411	0.363	2.260	1.120	0.361	0.288	1.952
ethyl hexadecanoate	1.276	0.639	-0.198	2.751	1.123	0.557	-0.162	2.409
3-methylbutanoic acid	1.276	0.349	0.471	2.080	1.107	0.307	0.399	1.816
propanoic acid	1.263	0.343	0.473	2.052	1.132	0.308	0.421	1.843
ethyl acetate	1.210	0.693	-0.387	2.808	1.193	0.447	0.162	2.224
ethyl levulate	1.101	0.650	-0.398	2.599	1.028	0.632	-0.430	2.486
ethyl vanillate	1.031	0.717	-0.621	2.684	1.161	0.381	0.283	2.039
(E)-whiskeylactone	1.019	0.502	-0.139	2.176	0.873	0.459	-0.186	1.932
β-damascenone	1.014	0.486	-0.108	2.136	0.928	0.498	-0.221	2.077
2-acetylfuran	0.933	0.764	-0.828	2.695	1.195	0.412	0.245	2.145
2-phenylethanol	0.932	0.673	-0.620	2.483	1.141	0.617	-0.283	2.564
vanillin	0.919	0.696	-0.687	2.524	1.147	0.337	0.370	1.924
2-methoxy-4-vinylphenol	0.727	0.707	-0.904	2.357	1.076	0.466	0.001	2.152
syringol	0.353	0.715	-1.295	2.000	1.080	0.535	-0.154	2.315

Variable	VIP	Standard deviation	Lower bound(95%)	Upper bound(95%)	VIP	Standard deviation	Lower bound(95%)	Upper bound(95%)
guaiacol	0.128	0.599	-1.253	1.509	1.051	0.519	-0.146	2.249

Supplementary Table 5 Variable Identification (VID) Coefficients of Selected Aroma Compounds for Each Aroma Attribute

Compounds	Alcohol	Dried fruit	Floral	Fruity	Caramel	Toasted	Spicy	Mushroom
(Z)-whiskeylactone		0.876						
1-hexanol							0.891	
2-acetylfuran	-0.847					0.892		
2-methyl-1-propanol	-0.857							
5-methylfurfural		0.825		-0.809			0.813	
benzaldehyde								0.907
ethyl acetate		0.870				0.932		
ethyl lactate		0.909	-0.837	-0.803			0.841	
ethyl octanoate								
ethyl vanillate						0.890		
furfural				-0.825			0.900	
octanoic acid								
vanillin						0.916		
β-damascenone								0.843
γ-dodecalactone			0.883	0.874			-0.834	
γ-nonalactone			0.906	0.864			-0.832	

	sample	subject	repeat.	sample:subject	sample:repeat.	subject:repeat.
floral	1.215e-42	0.01534	0.04197	0.7736	0.181	0.7797
toasted	2.722e-37	0.147	0.06966	0.5442	0.3035	0.5499
dried.fruit	3.632e-34	0.08164	0.7905	0.6002	0.1962	0.3673
alcohol	8.189e-34	0.001845	0.9076	0.1356	0.2549	0.2483
fruity	1.881e-33	0.1406	0.5417	0.9427	0.7451	0.4823
spicy	4.681e-33	0.007568	0.3953	0.2599	0.1888	0.08484
mushroom	1.786e-29	0.6353	0.2474	0.2515	0.9867	0.909
caramel	6.591e-28	1.446e-05	0.7175	0.01743	0.9081	0.04556

Supplementary Figure 1 Panel Performance in Evaluating Different Aroma Descriptor. The panel performance was assessed by ANOVA (*panelperf* function from the *SensoMineR* package (Husson & Le, 2008)), and statistically significant values ($p < 0.05$) were marked in the gray table cell. The dependent variables were the eight attributes and the independent variables were the factors associated with the sample effect, the subject effect, the repeat effect, and all their first-order interactions. If there was a significant sample effect, the panel discriminated the samples with respect to the target sensory attribute (discrimination); if there was a significant sample × subject interaction, subjects did not have the same perception of the samples with respect to the target sensory attribute (no agreement); if there was a significant sample × repeat interaction, subjects were not consistent among replications.
